# The Anti-Inflammatory Effects of Acupuncture and Their Relevance to Allergic Rhinitis: A Narrative Review and Proposed Model

**DOI:** 10.1155/2013/591796

**Published:** 2013-02-14

**Authors:** John L. McDonald, Allan W. Cripps, Peter K. Smith, Caroline A. Smith, Charlie C. Xue, Brenda Golianu

**Affiliations:** ^1^School of Medicine and Griffith Health Institute, Griffith Health, Griffith University, Gold Coast Campus, Southport, QLD 4215, Australia; ^2^Centre for Complementary Medicine Research, University of Western Sydney, Sydney, NSW 2751, Australia; ^3^Health Innovations Research Institute and WHO Collaborating Centre for Traditional Medicine, School of Health Sciences, RMIT University, Melbourne, VIC, Australia; ^4^Stanford University, Palo Alto, CA, USA

## Abstract

Classical literature indicates that acupuncture has been used for millennia to treat numerous inflammatory conditions, including allergic rhinitis. Recent research has examined some of the mechanisms underpinning acupuncture's anti-inflammatory effects which include mediation by sympathetic and parasympathetic pathways. The hypothalamus-pituitary-adrenal (HPA) axis has been reported to mediate the antioedema effects of acupuncture, but not antihyperalgesic actions during inflammation. Other reported anti-inflammatory effects of acupuncture include an antihistamine action and downregulation of proinflammatory cytokines (such as TNF-**α**, IL-1**β**, IL-6, and IL-10), proinflammatory neuropeptides (such as SP, CGRP, and VIP), and neurotrophins (such as NGF and BDNF) which can enhance and prolong inflammatory response. Acupuncture has been reported to suppress the expression of COX-1, COX-2, and iNOS during experimentally induced inflammation. Downregulation of the expression and sensitivity of the transient receptor potential vallinoid 1 (TRPV1) after acupuncture has been reported. In summary, acupuncture may exert anti-inflammatory effects through a complex neuro-endocrino-immunological network of actions. Many of these generic anti-inflammatory effects of acupuncture are of direct relevance to allergic rhinitis; however, more research is needed to elucidate specifically how immune mechanisms might be modulated by acupuncture in allergic rhinitis, and to this end a proposed model is offered to guide further research.

## 1. Introduction

Worldwide, allergic rhinitis is estimated to affect 18% of 15–34-year olds and 10% of 35–54-year olds [1]. Studies estimate that seasonal allergic rhinitis affects approximately from 10% to 20% of the general population of the United States of America (USA), with an even greater prevalence in children [2, 3]. An estimated 30 to 60 million people annually in the USA suffer from allergic rhinitis [2].

While the term “rhinitis” implies inflammation of nasal mucus membranes, clinically rhinitis can refer to any nasal disorder which includes any one or more of the symptoms: sneezing, nasal pruritus, rhinorrhea, and nasal congestion [2]. Rhinitis can be allergic (triggered by contact with an inhaled allergen) or nonallergic [4]. Allergic rhinitis is the most common form of chronic rhinitis, however, up to 87% of patients with allergic rhinitis also react to triggers which are not allergens such as cold air, perfumes, and smoke [2].

Links between allergic rhinitis and asthma have been highlighted by the allergic rhinitis and its impact on asthma (ARIA) group which recommends that allergic rhinitis and asthma should be regarded as related phenomena of airway reactivity and managed using a “united airway approach” [3, 5, 6].

While there is evidence that acupuncture treatment is clinically beneficial for patients with allergic rhinitis, little is currently understood about the mechanisms of acupuncture in this, or other chronic inflammatory diseases which involve changes in either the systemic or mucosal immune response. This paper will address the current state of research into the effects of acupuncture on the immune system with emphasis on anti-inflammatory actions, and specifically on effects on mucosal immunity in allergic rhinitis. Based on this paper, a model that hypothesizes the potential anti-inflammatory mechanisms of acupuncture for allergic rhinitis is proposed to guide future investigation.

## 2. Search Strategy

Two searches were conducted. The first search investigated the pathophysiology of allergic rhinitis with an emphasis on the roles of cytokines, proinflammatory neuropeptides, and neurotrophins. The second search identified the acupuncture research on allergic rhinitis and the anti-inflammatory actions of acupuncture, especially in allergic inflammatory response. Database searches were conducted using Medline, PubMed, ScienceDirect, EbscoHost, Wiley Online library, Cochrane Database of Controlled Trials and the search terms “acupuncture,” “allergic rhinitis,” “inflammation,” “anti-inflammatory,” “neurotrophin,” “neuropeptide,” “cytokine,” “substance P,” “SP,” “calcitonin gene-related peptide,” “CGRP,” “vaso-active intestinal peptide,” “VIP,” “histamine,” “TRPV1.” In addition the following journals were hand searched: Acupuncture Research (Zhen Ci Yan Jiu) (1984–2010), World Journal of Acupuncture-Moxibustion (1992–2011), Journal of Traditional Chinese Medicine (English edition) (1981–2011), Journal of Acupuncture and Tui Na Science (2010), and American Journal of Acupuncture (1973–1999).

## 3. Physiological and Immune Mechanisms of Allergic Rhinitis

Allergic rhinitis manifests as an allergic inflammatory response, an IgE-mediated reaction involving a complex interaction between inflammatory cells including eosinophils and mast cells, their released inflammatory cytokines, proinflammatory neuropeptides which promote vasodilation and plasma extravasation and neurotrophins which prolong survival of inflammatory cells and contribute to hypersensitivity [4]. Disruption of the integrity of the nasal epithelium through cleaving of tight junctions by protease activities (due to inflammation or airborne allergens) exposes sensory nerve endings, which enhances the neurogenic inflammatory response, especially the release of substance P (SP) and calcitonin gene-related peptide (CGRP) [4].

**Figure 1:**
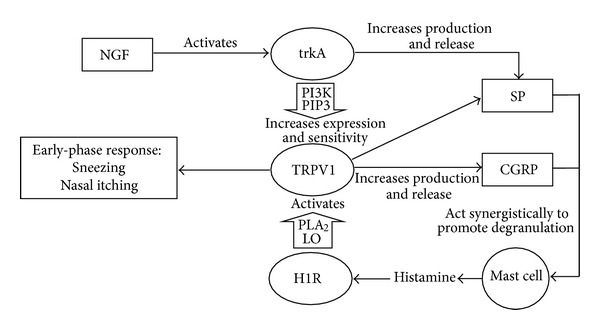
The role of transient receptor potential vallinoid 1 (TRPV1) in early-phase response in allergic rhinitis. Nerve growth factor (NGF) activates tyrosine kinase A (TrkA) receptor which in turn increases production and release of substance P (SP). Activation of TrkA receptor also initiates signalling via the PI3K/PIP3 pathway to increase expression and sensitivity of transient receptor potential vallinoid (TRPV1) receptor. TRPV1 receptor increases production and release of proinflammatory neuropeptides SP and CGRP which act synergistically to promote degranulation of primed mast cells. Histamine released by mast cells activates Histamine 1 receptor (H1R) producing signalling via the phospholipase A_2_/lipoxygenase pathway to activate TRPV1, triggering early-phase allergic inflammatory response. NGF: nerve growth factor, TRPV1: transient receptor potential vallinoid 1, TrkA: tyrosine kinase A receptor, H1R: histamine 1 receptor, SP: substance P, CGRP: calcitonin gene-related peptide, PI3K/PIP3: phosphatidylinositol 3 kinase/phosphatidylinositol phosphate 3 pathway, PLA_2_/LO: phospholipase A_2_/lipoxygenase pathway.

The early-phase allergic response in allergic rhinitis is triggered within minutes of allergen inhalation when IgE antibody, bound to mast cells, recognizes allergens and causes degranulation and release of inflammatory mediators such as histamine, tryptase, leukotrienes, prostaglandin D_2,_ and proinflammatory cytokines such as tumour necrosis factor alpha (TNF*α*) and interleukin 4 (IL-4) [4]. This early-phase response is generally characterized by sneezing, nasal itching, and rhinorrhoea [2, 4]. Sneezing and nasal itching have been shown to be neural responses mediated by histamine activating the histamine receptor H1R and the transient receptor potential vallinoid 1 (TRPV1) [7–10] (see Figure 1). Rhinorrhoea is primarily a glandular response involving nasal epithelial cells but also has neural and vascular contributions [7, 11]. Plasma extravasation, and vasodilation caused by mediators such as leukotrienes, prostaglandin D_2_, nitric oxide, and proinflammatory neuropeptides such as SP, CGRP, and vasoactive intestinal peptide (VIP) create nasal congestion [4, 12]. Kaise et al. found that, in guinea pigs, SP and CGRP released from nasal sensory nerves, possibly stimulated by mast cell-derived histamine, partially mediate the early-phase response [13]. Neurotrophin nerve growth factor (NGF) has been shown to contribute to early-phase response in allergic airway response but not to late-phase response in rats with allergic asthma [14]. 

Late-phase allergic response occurs from 4 to 8 hours after the initial early-phase response as cytokines and other inflammatory mediators set off a cascade of events which promotes the expression of adhesion molecules (which in turn increase the adhesion of eosinophils to endothelial cells) and promotes infiltration by eosinophils, basophils, and neutrophils into the superficial lamina propria of the nasal mucosa [4]. The symptoms of late-phase response are similar to those of early-phase response but with a greater predominance of nasal congestion [4]. SP, CGRP, and neurokinin A (NKA) (and their respective receptors NK-1, CGRP1, and NK-2) are reportedly involved in the late-phase response in allergic nasal obstruction in guinea pigs [13].

The release of proinflammatory cytokines such as TNF*α* and IL-4 from degranulated mast cells promotes the differentiation of CD4+ T helper (Th) cells into Th2 phenotype. This Th2 response promotes the production of eosinophils and the phenotype switching of B lymphocytes leading to increased production of IgE and increased proliferation and activation of mast cells [15]. The weighting of Th1/Th2 balance towards Th2 characterizes the allergic response [15].

### 3.1. The Role of Neuropeptides in Airway Inflammation

Nonopioid proinflammatory neuropeptides contribute to neurogenic inflammation by promoting vasodilation and plasma extravasation, notably in the nasal mucosa in allergic rhinitis [11]. The nasal mucosa has dense networks containing the proinflammatory neuropeptides SP, VIP, and CGRP which can arise from sensory and autonomic nerve fibres and from neuroendocrine cells found widely in the nasal mucosa [16]. SP has also been shown to be colocalized with TRPV1 and the neurotrophin receptor tyrosine kinase A (trkA) in airway-specific murine dorsal root ganglionic neurons [17]. In cultured rat trigeminal ganglionic neurons SP and CGRP were found to be colocalized with TRPV1, and also with three SNARE complex proteins: synaptobrevin 1, syntaxin 1 and SNAP 25, which mediate the exocytosis of CGRP from sensory neurons [18]. TRPV1 receptor activation mediates the production and exocytotic release of SP and CGRP from sensory neurons [17, 19]. 

In allergic airway inflammation (including allergic asthma and allergic rhinitis) SP and CGRP levels in the saliva and nasal secretions are elevated [16, 20]. SP in the nasal mucosa of humans increases eosinophil accumulation during repeated allergen exposure in allergic rhinitis [21]. SP and CGRP both activate monocytes to release the proinflammatory cytokines: TNF-*α*, IL-1*β*, IL-6, and IL-10 [22, 23]. The manner in which neuropeptides such as SP are able to modulate B-cell function is dependent on the activation of T cells by immunoregulatory cytokines such as IL-5 and TGF*β* [24]. SP has also been reported to upregulate the expression of macrophage inflammatory protein 1*β* (MIP-1*β*) in human T lymphocytes (*in vitro*) [25]. SP and CGRP often act synergistically and also potentiate each other in inflammatory oedema, in plasma extravasation during airway inflammation and in mast cell degranulation [26–28] (see Table 1). NGF activation of the high-affinity NGF receptor trkA can generate production and release of SP, while SP, in turn, can promote the production and release of NGF [17, 20, 29]. NGF can also promote CGRP content and release from TRPV1-expressing trigeminal ganglion neurons *in vitro* [30] (Figure 1).

**Figure 2:**
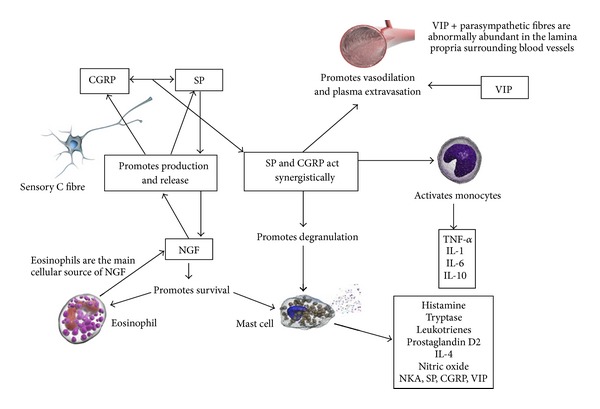
Complex crosstalk between inflammatory cells, neuropeptides, neurotrophins, and cytokines in allergic rhinitis. Substance P (SP) and calcitonin gene-related peptide (CGRP) act synergistically (along with vasoactive intestinal peptide (VIP)) to promote vasodilation and plasma extravasation causing nasal congestion. SP and CGRP also activate monocytes to release proinflammatory cytokines and promote degranulation of primed mast cells contributing to early-phase allergic response. Nerve growth factor (NGF) promotes the production and release of SP and CGRP and also promotes the survival of eosinophils and mast cells hence prolonging inflammatory response. CGRP: calcitonin gene-related peptide, SP: substance P, VIP: vasoactive intestinal peptide, NKA: neurokinin A, NGF: nerve growth factor, TNF-*α*: tumour necrosis factor alpha, IL-1: interleukin 1, IL-4: interleukin 4, IL-6: interleukin 6, IL-10: interleukin 10. Images courtesy of Dr P. K. Smith.

From this evidence it can be seen that proinflammatory neuropeptides such as SP, CGRP, and VIP interact with various immune cells including T lymphocytes, B lymphocytes, macrophages, monocytes, and mast cells to modulate allergic inflammation of the nasal mucosa. These interactions influence the release of cytokines and are capable of modifying Th1/Th2 balance in CD4+ T-cell differentiation. Proinflammatory neuropeptides can act synergistically and potentiate each other. Proinflammatory neuropeptides and neurotrophins can promote each other's production and release, creating a positive feedback cycle (Figure 2).

### 3.2. The Role of Neurotrophins in Airway Inflammation

Neurotrophins, or nerve growth factors, are proteins which regulate the survival, death, or differentiation of neurons. The primary function of neurotrophins is to promote nerve growth. The main categories of neurotrophins include NGF, brain-derived neurotrophic factor (BDNF), glial cell-derived neurotrophic factor (GDNF), neurotrophin 3 (NT-3), and neurotrophin 4/5 (NT 4/5). The density of innervation to the nasal mucosa in allergic rhinitis patients is reported to be double that of healthy individuals [16, 31, 32]. Much of this additional innervation surrounds arterial blood vessels in the lamina propria and principally involves VIP-containing parasympathetic nerve fibres [32–34]. In allergic rhinitis this neuronal abundance is likely to contribute to hypersensitivity as well as to amplifying the allergic inflammatory response. Concentrations of NGF, BDNF, and NT-3 increase dramatically in the respiratory epithelium during allergic rhinitis [20, 35]. Both nasal NGF and BDNF expressions were reported to be significantly increased in allergic rhinitis patients compared to healthy controls after nasal allergen provocation [36]. This allergen-induced increase in BDNF correlated with the maximal increase in total nasal symptom score (TNSS), suggesting an important role for this neurotrophin in modifying symptom severity in allergic rhinitis patients [36].

In addition to promoting neuronal proliferation, neurotrophins also prolong the survival of eosinophils and mast cells, thereby prolonging the inflammatory response [20]. Eosinophils, mast cells, monocytes and macrophages, in turn, all produce NGF [20, 37, 38]. Wu et al. reported that the major sources of NGF in the human nasal mucosa are submucosal glands and nasal epithelium, with eosinophils being the major cellular source (while mast cells account for only a small fraction) [38] (Table 2).

These findings suggest not only an important role for SP, CGRP, and VIP in promoting and amplifying allergic airways inflammation, but also suggest a complex interaction between inflammatory cells, cytokines, and neurotrophins with these proinflammatory neuropeptides (Figure 2).

### 3.3. The Role of TRPV1 Receptor in Early-Phase Allergic Inflammatory Response

TRPV1 receptor is a polymodal receptor which is activated by several triggers including capsaicin, noxious heat (42–53°C), low extracellular pH, ethanol, acids, pollution, protons, and lipids [17, 39]. TRPV1-positive cells are found on epithelial cells, vascular endothelial cells, submucosal glands, and nerves in human nasal mucosa [40]. TRPV1 has been shown to be colocalized with the neurotrophin receptor tyrosine kinase trk-A and SP in airway-specific murine dorsal root ganglionic neurons [17]. In cultured rat trigeminal ganglionic neurons, TRPV1 was found to be colocalized with SP, CGRP, and the SNARE complex proteins synaptobrevin 1, syntaxin 1 and SNAP 25 (which mediate the exocytosis of CGRP) [18]. The expression and sensitivity of TRPV1 receptor can be upregulated by NGF-induced activation of trkA receptor which signals via the PI3K/PIP3 pathway [41] (see Figure 1). TRPV1 increases the production and exocytotic release of proinflammatory neuropeptides SP and CGRP which act synergistically to promote the degranulation of primed mast cells [17].

Histamine is generally regarded as the archetypal mediator of allergic inflammatory response. Histamine released by degranulating mast cells activates the histamine 1 receptor (H1R) which in turn activates the TRPV1 receptor via the phospholipase A_2_/lipoxygenase (PLA_2_/LO) pathway [8]. Histamine-induced activation of TRPV1 triggers the early-phase response in allergic rhinitis [8]. These pathways are outlined in Figure 1.

## 4. Clinical Efficacy and Effectiveness of Acupuncture for the Treatment of Allergic Rhinitis

Two early systematic reviews concluded that there was insufficient evidence to demonstrate the efficacy of acupuncture in allergic rhinitis, however, these reviews were limited by the paucity and quality of studies available (3 and 7 studies up to 2004) [42, 43]. A more recent and more comprehensive systematic review (12 studies up to 2008 involving 1076 patients) concluded that acupuncture and moxibustion were safe and effective to treat allergic rhinitis and may have some advantages over routine medication [44]. Another recent systematic review (12 studies up to 2007) found suggestive evidence for the effectiveness of acupuncture in persistent allergic rhinitis, but no significant difference between real and sham acupuncture for seasonal allergic rhinitis [45]. This may be due to relatively few studies being published to date on seasonal allergic rhinitis.

Since publication of the most recent review, evidence of benefit has been further supported by findings from a large trial which included two randomized groups and one nonrandomized group, involving 5237 patients with allergic rhinitis. Brinkhaus and colleagues found that when acupuncture was added to routine medical care, there were statistically and clinically relevant benefits [46]. Rhinitis Quality of Life Questionnaire (RQLQ) scores after 3 months of acupuncture treatment improved by a mean (SE) of 1.48 (0.06) in the acupuncture group, by 0.50 (0.06) in the control group, with a difference in the improvement of 0.98 (0.08) (*P* < 0.001) [46].

## 5. Mechanisms by Which Acupuncture May Moderate the Clinical Symptoms of Allergic Rhinitis

### 5.1. Overview of Possible Anti-Inflammatory Mechanisms of Acupuncture

Recent research has elucidated some of the mechanisms underpinning acupuncture's anti-inflammatory effects. Multiple physiological pathways appear to mediate the anti-inflammatory effects of acupuncture including the hypothalamus-pituitary-adrenal (HPA) axis [47–50], sympathetic pathways (via both sympathetic postganglionic neurons and the sympathoadrenal medullary axis) [49, 50], and possibly parasympathetic cholinergic pathways [51–54].

Other relevant anti-inflammatory effects of acupuncture include antihistamine effects [55–58] and downregulation of proinflammatory cytokines (such as TNF-*α*, IL-1*β*, IL-6, and IL-10) [59–65], and proinflammatory neuropeptides (such as SP, CGRP, and VIP) [66, 67]. The involvement of both opioid and nonopioid neurotransmitters has been demonstrated [68–73]. Neurotrophins (such as NGF, BDNF, and NT-3) which contribute to hypersensitivity, as well as enhance and prolong inflammatory response, have been shown to be downregulated by acupuncture [74–78]. Acupuncture has also been found to suppress the expression of COX-1, COX-2, and iNOS during experimentally induced inflammation [79]. NMDA and AMPA/KA receptors (receptors for glutamate and aspartate) have also been implicated in the anti-inflammatory actions of acupuncture [80, 81]. The effects of acupuncture on TRPV1 have also been examined. Further research is needed to clarify the role these anti-inflammatory actions of acupuncture may play in the context of treatment for allergic rhinitis.

### 5.2. The HPA Axis

Acupuncture effects on the inflammatory response have been shown to be modulated by the HPA axis in a number of animal studies. In a carrageenan-induced paw inflammation rat model, the antioedema effects of electroacupuncture were abolished by various disruptions of the HPA axis including adrenalectomy and antagonizing receptors for corticotropin releasing hormone (CRH), adrenocorticotropic hormone (ACTH), or glucocorticoids [47, 48]. The involvement of the HPA axis in the anti-inflammatory effects of acupuncture was further supported by findings of significant increases in levels of ACTH and corticosterone in the same rat paw inflammation model in response to electroacupuncture [47, 48]. However, the disruption of the HPA axis has been found to have no effect on the antihyperalgesic effects of acupuncture or electroacupuncture suppression of leucocyte migration (in a mouse air pouch inflammation model) [47–49]. The HPA axis-mediated acupuncture inhibition of inflammatory oedema may be involved in the reduction of nasal congestion in allergic rhinitis.

### 5.3. Sympathetic Pathways

Leucocyte migration appears to be mediated by the activation of *β*-2 adrenoreceptors on leucocytes by noradrenalin released from sympathetic postganglionic neurons in response to low-frequency electroacupuncture [49, 50]. Low-frequency electroacupuncture leads to the suppression of carageenan-induced paw oedema in mice which appeared to be mediated via sympathetic postganglionic neurons [50]. Conversely, high-frequency electroacupuncture also had significant anti-inflammatory effects, this time mediated via the sympathoadrenal medullary axis [50]. Sympathetic mediation of acupuncture inhibition of inflammatory oedema may also be involved in alleviating nasal congestion in allergic rhinitis.

### 5.4. Parasympathetic Cholinergic Pathways

A parasympathetic anti-inflammatory pathway mediated by acetylcholine (ACh) has been demonstrated in research not related to acupuncture. ACh released by the vagus nerve binds to *α*7-nicotinic receptors (*α*7nAChR) on macrophages which inhibits the release of proinflammatory cytokines [51–54]. It has been proposed that this cholinergic anti-inflammatory pathway may be activated by acupuncture; however, no direct experimental confirmation of this hypothesis is currently available [51].

### 5.5. Antihistamine Action

Acupuncture has been reported to reduce histamine-induced itch in healthy subjects [55–57]. Prophylactic acupuncture (for 15 minutes prior to topical histamine application to the skin) was shown to significantly reduce histamine-induced itch and wheal formation in healthy subjects, compared with placebo-point acupuncture and no intervention [57]. Type I hypersensitivity itch, wheal, and flare response to allergen challenge in patients with atopic eczema was also significantly reduced by acupuncture [58].

A possible mechanism for an antihistamine action of acupuncture may be the downregulation of signalling in TRPV1 receptors, which mediate histamine-induced symptoms of allergic rhinitis such as nasal itching, sneezing, and rhinorrhoea [8, 10].

### 5.6. Cytokines

Changes in cytokines which would be expected to be associated with an improvement in allergic inflammation include downregulation in Th2 cytokines such as IL-4, IL-6, and IL-10 and proinflammatory cytokines such as IL-1, IL-6, and IL-10 accompanied by an upregulation in Th1 cytokines such as IL-2 and IFN-*γ*. 

Some evidence of a shift in Th1/Th2 balance away from Th2 has been shown in studies of acupuncture treatment of allergic rhinitis in humans, namely, a significant reduction in IL-10 and IL-4 and a significant decrease in gene expression for IL-1R1 [59–61] (see Table 3). Rao and Han reported no change in IFN-*γ* in humans with allergic rhinitis; however, in another recent study, Zheng et al. did report a significant increase in IFN-*γ* together with a significant decrease in the Th2 cytokines IL-4 and GM-CSF (granulocyte-macrophage colony stimulating factor) [60, 62]. After two courses of 15-second-daily acupuncture treatments, IL-4 and GM-CSF decreased while IFN-*γ* increased (*P* < 0.01) until the levels of all three cytokines in peripheral blood were similar to those of the healthy controls [62].

Immediately after a single acupuncture treatment, Petti et al. reported a significant decrease in IL-10, no significant change in IL-6, and an unexpected significant decrease in IL-2 [59]. Since the effects of a single acupuncture treatment are not likely to accurately predict the effects of a substantial course of acupuncture, it is difficult to interpret the results of this study.

In other inflammatory conditions, studies measuring the effects of acupuncture on Th2 and proinflammatory cytokines have reported significant reductions in IL-1*β* and TNF-*α* in carrageenan-induced hind paw inflammation in rats [63]. Acupuncture has also been found to significantly reduce IL-6 and IL-10 in humans with asthma [64]. In a rodent model of experimental asthma, electroacupuncture increased IL-1 and IFN*γ* and decreased IL-4, IL-10, nitric oxide, and leukotriene B4 in bronchoalveolar lavage and pulmonary tissue compared with control and sham acupuncture groups [65]. Secretion of Th2 promoting cytokines IL-4 and IL-13 was suppressed after acupuncture in a study using DNP-KLH immunized mice [82].

### 5.7. Neuropeptides

Studies on the effects of acupuncture on neuropeptides can be divided broadly into research on opioid neuropeptides and nonopioid proinflammatory neuropeptides.

#### 5.7.1. Opioid Neuropeptides

Much of the research on acupuncture suppression of inflammatory hyperalgesia overlaps with the broader research on acupuncture's antinociceptive actions and the role of opioid neuropeptides in these effects. Opioid neuropeptides which have been shown to mediate antinociceptive effects of acupuncture include enkephalins, *β*-endorphin, endomorphins, dynorphins, and nociceptin/orphanin FQ, and different frequencies of electroacupuncture have been shown to stimulate the production and release of different neuropeptides in a highly selective manner [83].

In animal experiments, intraperitoneal injection of Naloxone, a generic opioid receptor antagonist, has been shown to significantly decrease electroacupuncture suppression of hyperalgesia and leucocyte migration [69, 84]. However electroacupuncture's effects in reducing oedema in a carageenan-induced hind paw inflammation model were unaffected by intraperitoneal Naloxone [68]. This finding is consistent with other studies which have shown that the antioedema effects of lowfrequency electroacupuncture are mainly mediated via the HPA axis and sympathetic postganglionic neurons, rather than via central or peripheral opioid pathways [47, 48, 50].

Another possible role for opioid neuropeptides in acupuncture for allergic rhinitis is the inhibition of non-opioid proinflammatory neuropeptides such as SP. The opioid neuropeptide enkephalin inhibits or regulates SP release from peripheral nerve endings via the activation of opiate receptors, suggesting a possible role for enkephalin in the downregulation of SP by acupuncture [67, 71, 72]. 

Opioid receptors have been identified on numerous types of immune cells including B-lymphocytes, T-lymphocytes, natural killer cells, granulocytes, and monocytes, however the role of endogenous opioid neuropeptides in the anti-inflammatory effects of acupuncture has yet to be elucidated [73].

#### 5.7.2. Nonopioid Proinflammatory Neuropeptides (SP, CGRP, VIP)

Only one study has examined the effects of acupuncture on the proinflammatory neuropeptides SP and VIP in humans with allergic rhinitis [66]. SP and VIP were measured in plasma from venous blood using radioimmunoassay. When one group receiving 30 treatments of electroacupuncture was compared to another group receiving medication (Cetirizine 10 mg three times daily), both groups showed a significant lowering of both SP and VIP after treatment compared to pretreatment [66]. The electroacupuncture group had a significantly greater reduction in VIP than the medication group, but there was no significant difference between groups in reduction of SP [66]. Decreased levels of SP and VIP were also closely correlated with improvements in clinical signs and symptoms [66]. 

The topical application of Chinese herbal paste to acupuncture points has also been reported to inhibit celiac mast cell degranulation in mice with allergic rhinitis [85]. These findings suggest that the inhibition of mast cell degranulation may be one of the important clinical effects of acupuncture in the early-phase response in allergic rhinitis and that this inhibition may be achieved, in part, by downregulation of proinflammatory neuropeptides such as SP, CGRP, and VIP (which have been shown to promote mast cell degranulation) [26–28].

### 5.8. Neurotrophins, IgE and Eosinophils

Acupuncture has been reported to upregulate and downregulate neurotrophins; however, to date there have been no studies measuring the effects of acupuncture in allergic rhinitis [77]. The reported effects of acupuncture on neurotrophins, IgE and eosinophils are summarized in Table 4.

### 5.9. Clinical Outcome Measurements: Nasal Congestion and Nasal Ciliary Clearance Rates

Acoustic rhinometry after a single acupuncture treatment for allergic rhinitis was used to measure nasal volume (cm^3^) (NV) and total nasal minimal cross-sectional area (cm^2^) (MCA) [93]. A statistically significant increase in NV and MCA was reported immediately after acupuncture in both active and placebo acupuncture groups, with a greater increase in the active acupuncture group. In the active acupuncture group, increases in NV and MCA persisted for 15 minutes but were not significant after 7.5 minutes, while in the placebo group NV dropped below baseline after 7.5 minutes and MCA was also below baseline after 15 minutes. This suggests that a single acupuncture treatment has an immediate but very short-lived effect in decreasing nasal congestion in humans with allergic rhinitis.

In a prospective pragmatic open study involving 45 patients with allergic rhinitis, when acupuncture was compared to antihistamine medication over a seven-week period, nasal ciliary clearance rates increased significantly in both groups but were faster in the acupuncture group, both immediately after treatment and at 3-month followup [92].

### 5.10. The Effects of Acupuncture on TRPV1 Receptor in Early-Phase Allergic Inflammatory Response

Acupuncture has been shown to inhibit TRPV1 signalling, but the mechanism for this action remains unclear [94]. In a cancer pain model, electroacupuncture has been shown to suppress TRPV1 mRNA and protein upregulation in the dorsal root ganglia of tumour-bearing rats [94]. In an inflammatory pain model, the acupuncture-induced activation of A1 receptors by adenosine has been shown to be essential to the antinociceptive effects of manual acupuncture [95]. Adenosine can directly inhibit TRPV1 activation [96]. Another possible pathway is the downregulation of NGF activation of tyrosine kinase receptor trkA which uses the phosphatidylinositol 3-kinase/phosphatidylinositol phosphate 3 (PI3K/PIP3) signalling pathway to increase the expression and sensitivity of TRPV1 [41]. In an inflammatory pain model, electroacupuncture has recently been shown to inhibit phosphorylation of spinal PI3K, hence preventing the production of PIP3 and downstream protein kinase Akt [97]. This demonstrates that electroacupuncture is capable of blocking the PI3K/PIP3 signalling pathway which is essential to NGF-induced enhancement of TRPV1 expression and sensitivity.

In summary, acupuncture may inhibit TRPV1 expression and sensitivity by downregulating the production and release of NGF and/or by blocking PI3K/PIP3 signalling between trkA receptor and TRPV1. Acupuncture-induced inhibition of TRPV1 may be achieved by downregulating SP and CGRP which in turn would reduce degranulation of mast cells thereby reducing histamine release and histamine activation of TRPV1 via H1R. Another possibility is that downregulation of TRPV1 (regardless of the source of this downregulation) causes the reduction in SP and CGRP release, or perhaps this is a negative feedback loop. Acupuncture inhibition of TRPV1 may also involve adenosine release. 

## 6. A Proposed Model for the Mechanism of Acupuncture in Allergic Rhinitis

**Figure 3:**
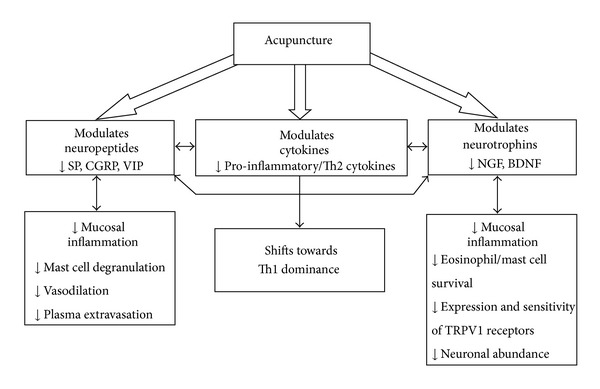
Proposed model for the effects of acupuncture in mucosal inflammation. CGRP: calcitonin gene-related peptide, SP: substance P, VIP: vasoactive intestinal peptide, NGF: nerve growth factor, BDNF: brain-derived neurotrophic factor, TRPV1: transient receptor potential vallinoid 1.

Given the complex crosstalk between cytokines, neuropeptides and neurotrophins in allergic inflammation, it is hypothesized that acupuncture might exert anti-inflammatory actions in allergic rhinitis in three ways: firstly, by down-regulating Th2 and proinflammatory cytokines and up-regulating Th1 cytokines; secondly, by down-regulating proinflammatory neuropeptides (namely SP, VIP, and CGRP) and finally, by downregulating neurotrophins (NGF and BDNF) (see Figure 3). 

If acupuncture can be shown to have these actions on modulating cytokines, neuropeptides, and neurotrophins in allergic rhinitis, then these modulations would be expected to be correlated with improvements in clinical signs and symptoms, including a reduction in hyperresponsiveness, sneezing, nasal itching, rhinorrhea, and nasal congestion. Any reduction in Th2 dominance would also suggest a modulation of allergic status.

## 7. Conclusion

The role of neurotrophins in neurogenic inflammation, and in particular allergic airway inflammation, has recently been studied; however, the complex crosstalk between neurotrophins, neuropeptides, and cytokines in allergic airway inflammation is still poorly understood. Further studies are needed to elucidate some of the interactions between the neuropeptides: SP, CGRP, and VIP and the neurotrophins NGF and BDNF and some Th1, Th2, and proinflammatory cytokines in allergic rhinitis. 

Acupuncture has been reported to improve clinical outcomes in patients with allergic rhinitis, and some aspects of the anti-inflammatory actions of acupuncture have been studied. Little research to date has investigated the mechanisms by which acupuncture may modulate immune response in the upper airways to improve clinical outcomes in patients with allergic rhinitis. Although the actions of some neuropeptides in the antinociceptive effects of acupuncture have been extensively studied, the possible contribution that both opioid and non-opioid neuropeptides may make to inflammation, specifically allergic inflammation, has yet to be clarified. It is suggested in our theoretical model that, in adult subjects with allergic rhinitis, acupuncture may down-regulate certain proinflammatory neuropeptides and neurotrophins as well as Th2 cytokines and proinflammatory cytokines, thereby producing a shift in the Th1/Th2 balance of T helper cells towards Th1. Together these hypothesized actions would be expected to alleviate clinical signs and symptoms of allergic rhinitis. Further studies, guided by this model, are needed (using both animal and human models) to explore the effects of acupuncture at the cellular level on the inflammatory cascade described above, as well as the clinical effects of this treatment on symptomatic relief over time.

## Figures and Tables

**Table 1:** 

Role of SP and CGRP in allergic rhinitis:
(i) promote vasodilation and plasma extravasation in nasal epithelium (nasal congestion)
(ii) SP and CGRP act synergistically and potentiate each other in mast cell degranulation
(early-phase allergic response) and plasma extravasation (nasal congestion)
(iii) activate monocytes to release pro-inflammatory cytokines (early-phase allergic response)
(iv) increase eosinophil accumulation in nasal mucosa during repeated allergen exposure
(v) SP promotes production and release of NGF

**Table 2:** 

Role of NGF in allergic rhinitis:
(i) increases neuronal abundance in nasal epithelium leading to hypersensitivity and increased tendency to nasal congestion
(ii) increases expression and sensitivity of TRPV1 receptors in nasal epithelium
(iii) prolongs survival of eosinophils and mast cells (prolonging inflammatory response)
(iv) contributes to early-phase allergic response (but not to late-phase response)
(v) increases production and release of pro-inflammatory neuropeptides SP and CGRP

**Table 3:** Th1/Th2 cytokines in studies of acupuncture for allergic rhinitis.

Reference	Measurement method	Th2 cytokines				Th1 cytokines	
		IL-1*β*	IL-4	IL-10	GM-CSF	IFN-*γ*	IL-2
[59]	Peripheral blood plasma			↓			↓
[60]	Peripheral blood serum		↓			No change	
[61]	RNA from peripheral blood	↓					
[62]	Supernatant from peripheral blood monocytes		↓		↓	↑	

Reported increase: ↑, Reported decrease: ↓.

**Table:** (a) Neurotrophins: biphasic action depending on disease and model

Disease	Model	Reported effect	Reference
Parkinson's disease	Rodent	Upregulates BDNF in substantia nigra	[86]
Retinitis pigmentosa	Rodent	Upregulates NGF and BDNF in retina	[87]
Spinal cord injury	Feline	Upregulates NGF in spine	[88]
Polycystic ovarian syndrome (PCOS)	Rodent	Downregulates NGF in ovaries	[74–76]

**Table:** (b) Serum IgE: contradictory findings

Disease	Model	Reported effect	Reference
Allergic rhinitis	Human	No significant decrease	[60]
Allergic rhinitis	Human	No significant decrease	[89]
Allergic rhinitis	Human	No significant decrease	[90]
Allergic rhinitis	Human	Significant decrease	[91]

**Table:** (c) Eosinophils: contradictory findings

Disease	Model	Reported effect	Reference
Allergic rhinitis	Human	No significant difference in nasal or blood eosinophils	[90]
Allergic rhinitis	Human	No significant difference in blood eosinophils	[92]
Allergic rhinitis	Human	Significant decrease in blood eosinophils and percentage of nasal eosinophils	[91]
